# Temozolomide Treatment Induces HMGB1 to Promote the Formation of Glioma Stem Cells via the TLR2/NEAT1/Wnt Pathway in Glioblastoma

**DOI:** 10.3389/fcell.2021.620883

**Published:** 2021-02-01

**Authors:** Xiang-Yu Gao, Jian Zang, Min-Hua Zheng, Yu-Fei Zhang, Kang-Yi Yue, Xiu-Li Cao, Yuan Cao, Xin-Xin Li, Hua Han, Xiao-Fan Jiang, Liang Liang

**Affiliations:** ^1^State Key Laboratory of Cancer Biology, Department of Biochemistry and Molecular Biology, Fourth Military Medical University, Xi'an, China; ^2^Department of Neurosurgery, Xijing Hospital, Fourth Military Medical University, Xi'an, China; ^3^Department of Medical Genetics and Developmental Biology, Fourth Military Medical University, Xi'an, China; ^4^Institute of Medical Research, Northwestern Polytechnical University, Xi'an, China

**Keywords:** high mobility group box 1, glioma stem cell, TLR2, Wnt, NEAT1, temozolomide

## Abstract

Formation of glioma stem cells (GSCs) is considered as one of the main reasons of temozolomide (TMZ) resistance in glioma patients. Recent studies have shown that tumor microenvironment-derived signals could promote GSCs formation. But the critical molecule and underlying mechanism for GSCs formation after TMZ treatment is not entirely identified. Our study showed that TMZ treatment promoted GSCs formation by glioma cells; TMZ treatment of biopsy-derived glioblastoma multiforme cells upregulated HMGB1; HMGB1 altered gene expression profile of glioma cells with respect to mRNA, lncRNA and miRNA. Furthermore, our results showed that TMZ-induced HMGB1 increased the formation of GSCs and when HMGB1 was downregulated, TMZ-mediated GSCs formation was attenuated. Finally, we showed that the effect of HMGB1 on glioma cells was mediated by TLR2, which activated Wnt/β-catenin signaling to promote GSCs. Mechanistically, we found that HMGB1 upregulated NEAT1, which was responsible for Wnt/β-catenin activation. In conclusion, TMZ treatment upregulates HMGB1, which promotes the formation of GSCs via the TLR2/NEAT1/Wnt pathway. Blocking HMGB1-mediated GSCs formation could serve as a potential therapeutic target for preventing TMZ resistance in GBM patients.

## Introduction

Glioblastoma multiforme (GBM) is the most common primary brain tumor with the average survival of only about 15 months in patients receiving appropriate treatment (Stupp et al., 2005, 2009). The recognized treatment at present is surgical resection followed by adjuvant therapies such as radiotherapy and chemotherapy (Stupp et al., 2005). Temozolomide (TMZ) is one of the few medicines with a proven efficiency against GBM by inducing tumor cell death via methylating DNA (Ma et al., 2016). However, TMZ treatment also results in drug resistance, contributing to unsatisfactory prognosis for glioma patients. The mechanism of TMZ resistance reported so far is related to the heterogeneity of glioma cells, upregulation of O6-methylguanine DNA methyltransferase (MGMT), DNA repair, and signal transducer and activator of transcription 3 (STAT3) (Pajonk et al., 2010; Happold et al., 2012; Kohsaka et al., 2012).

Cancer stem-like cells (CSCs) are cancer cells that possess certain degree of stemness, including the ability to self-renew, proliferate, differentiate into more “mature” tumor cells with differentiated properties, and initiate tumorigenic process at high efficiency (Clarke, 2005; Batlle and Clevers, 2017). An important characteristic of CSCs is resistance to radiotherapy and chemotherapy (Olivares-Urbano et al., 2020; Sun et al., 2020; Walcher et al., 2020). Multiple studies have confirmed the existence of CSCs, or glioma stem cells (GSCs), in GBM (Lathia et al., 2015; Hira et al., 2018; Ma et al., 2018). GSCs constitute a rare cell subpopulation with stem cell characteristics in GBM, which are highly similar to neural stem cells (Ignatova et al., 2002; Singh et al., 2003). There is a consensus that GSCs are the main cause of tumor recurrence after chemotherapy with TMZ (Jiapaer et al., 2018). In addition, studies have reported that metabolites and cytokines secreted by tumor cells can regulate the tumor-initiating ability of GSCs and thus mediate resistance to TMZ (Calabrese et al., 2007; Li et al., 2009). However, the potential mechanism of GSCs formation and resistance to TMZ remains to be elucidated.

At present, the origin of CSCs is not yet fully understood. Recent studies have shown that tumor microenvironment (TME), which is composed of immune cells, perivascular cells, fibroblasts and factors secreted by these cells, can provide extracellular signals for the generation and maintenance of CSCs (Dzobo et al., 2020). It was recently reported that chemotherapy could cause immunogenic cell death (ICD) of tumor cells, which release damage-associated molecular patterns (DAMPs) into TME (Inoue and Tani, 2014). Secreted DAMPs as a result of ICD include high-mobility group box 1 (HMGB1), adenosine triphosphate (ATP), heat-shock proteins and calreticulin. HMGB1 is a highly conserved protein and expressed in many cell types (Sims et al., 2010). In the extracellular environment, HMGB1 can exert various biological functions by binding to high-affinity receptors including Toll-like receptor (TLR) 2, TLR4, TLR9, and the receptor for advanced glycation end-products (RAGE) (Angelopoulou et al., 2016). Additionally, HMGB1 derived from tumor cells or TME could promote the CSCs phenotype in lung, colon, pancreatic cancer cells (Zhao et al., 2017; Qian et al., 2019; Zhang et al., 2019). HMGB1 has also been reported to be upregulated in GBM and played a significant role in proliferation, apoptosis, migration, and invasion of GBM (Wang X. et al., 2015; Angelopoulou et al., 2016). Our recent study has shown that HMGB1 could promote the GSCs phenotype. However, the biological effects of HMGB1 on GSCs have not been studied in detail. In this study, we show that TMZ treatment upregulates HMGB1 in GBM cells *in vitro*. HMGB1 mediates the effect of TMZ in inducing the formation of GSCs via TLR2/NEAT1/Wnt/β-catenin signaling, thus might promote the resistance to TMZ in GBM patients.

## Materials and Methods

### Culture of Biopsy-Derived GBM Cells

Culture of biopsy-derived GBM cells has been described previously (Zang et al., 2020). Tumor tissues were collected from GBM patients accepting neurosurgery at Xijing Hospital, with signed informed consent and approved by the Ethics Committee of Xijing Hospital for use of human samples. Tumor tissues were dispersed and cultured in Dulbecco's modified Eagle's medium (DMEM)/F12 (1:1) (Invitrogen, Carlsbad, CA) supplemented with 10% fetal bovine serum (FBS, Invitrogen) and 1% penicillin–streptomycin solution. Cells were cultured for three passages and then frozen for further use. To culture GSCs, GBM cells were seeded in low adhesion plates (Corning Inc., Corning, NY) and cultured under the neurosphere condition in DMEM/F12 with 20 ng/mL epidermal growth factor (EGF, Peprotech, Rocky Hill, NJ), 10 ng/mL basic fibroblast growth factor (bFGF, Peprotech), B27 (1:50, Invitrogen), N2 (1:100, Invitrogen) and 1% penicillin–streptomycin solution for 7 days, and the number and size of tumor spheres were quantified. For re-plating, spheres were dispersed by Accutase (Invitrogen), counted, and cultured as above for 7 days. For differentiation, spheres were dissociated by Accutase into single cells and cultured in DMEM/F12 median supplemented with 10% FBS for 5 days. Cells were treated with TMZ (300 μM, MedChem Express, Monmouth Junction, NJ) for 48 h. In other cases, cells were treated with recombinant human HMGB1 (rhHMGB1, R&D Systems, Minneapolis, MN) at different concentrations (0, 200, 400, 600, 800, 1,000 ng/ml) for 48 h.

### Transfection of Cells With siRNA

siRNA against targeted genes and negative control siRNA (siCtrl) were designed and synthesized by RiboBio (Guangzhou, China). GBM cells were transfected with 10 nM of siRNA using Lipofectamine 2000 (Life Technologies) following the manufacturer's protocol. Cells were re-plated for tumor sphere assay 48 h after the transfection, or for RNA and protein extraction. The sequences of the siRNA used are listed as followed: HMGB1-siRNA1, 5′- GAGGCCUCCUUCGGCCUUC and 5′- GAAGGCCGAAGGAGGCCUC; HMGB1-siRNA2, 5′- GUUGGUUCUAGCGCAGUUU and 5′- AAACUGCGCUAGAACCAAC; TLR2-siRNA1, 5′-GCCCUCUCUACAAACUUUATT and 5′-UAAAGUUUGUAGAGAGGGCTT; TLR2-siRNA2, 5′-GCCUUGACCUGUCCAACAATT and 5′-UUGUUGGACAGGUCAAGGCTT; β-catenin-siRNA1, 5′- GACUACCAGUUGUGGUUAA and 5′- UUAACCACAACUGGUAGUC; β-catenin-siRNA2, 5′- GAUGGACAGUAUGCAAUGA and 5′- UCAUUGCAUAC; NEAT1-siRNA1, 5′- CGUCAGACUUGCAUACGCA and 5′- UGCGUAUGCAAGUCUGACG; NEAT1-siRNA2, 5′- GACCACUUAAGACGAGAUU and 5′- AAUCUCGUCUUAAGUGGUC.

### Immunofluorescence

Cells were fixed in 4% paraformaldehyde (PFA) for 10 min and blocked with 1% bovine serum albumin (BSA) for 30 min. Rabbit anti-HMGB1 (1:500, Abcam, Cambridge, UK), mouse anti-MAP2 (1:1,000, Sigma, St. Louis, MO), rabbit anti-GFAP (1:500, Sigma) and mouse anti-O4 (1:100, Sigma) were used as primary antibodies. Secondary antibodies included Cy2-conjugated donkey anti-mouse (1:500) and Cy2-conjugated donkey anti-rabbit (1:500, Jackson ImmunoResearch, West Grove, PA). Samples were examined under a fluorescence microscope (FV-100, Olympus, Japan).

### Flow Cytometry

Primary GBM cells were isolated and incubated with a PE anti-human CD133 (1:50, Biolegend, San Diego, CA) for 30 min at 4°C in dark. Then cells were analyzed by FACS using a FACS CaliburTM flow cytometer (BD Immunocytometry Systems, USA). Dead cells were excluded by propidium iodide (PI) staining. The acquired data were analyzed with FlowJo vX.0.6 software (Tree Star Inc., Ashland, OR).

### Enzyme-Linked Immunosorbent Assay (ELISA)

HMGB1 in culture supernatants was determined with an ELISA kit (Chondrex, Redmond, WA) according to the manufacturer's instructions.

### RNA-Sequencing (RNA-seq)

RNA-seq and data analyses were provided by commercial services (Gene Denovo Biotechnology, Guangzhou, China). RNA was extracted using the Trizol reagent (Thermo Fisher, Waltham, MA) and rRNA was removed. RNA samples were fragmented into appropriate short fragments, followed by reverse transcription with random hexamers, and second-strand cDNA synthesis. The cDNA fragments were purified with QiaQuick PCR extraction kit (Qiagen, Duesseldorf, Germany), end-repaired, and ligated to adapters. The uracil-N-glycosylase (UNG) was used to degrade the second-strand cDNA. The digested products were size-selected by agarose gel electrophoresis, PCR-amplified, and sequenced on Illumina HiSeq 3000 platform. Raw data of RNA-seq reported in this paper have been deposited in the Genome Sequence Archive in BIG Data Center (Beijing) under the accession number CRA003319, which is publicly accessible at https://bigd.big.ac.cn/gsa/.

For miRNA sequencing, RNA fragments of 18–30 nucleotides in length were enriched by polyacrylamide gel electrophoresis (PAGE). After adding 3' and 5' adapters, samples were subjected to RT-PCR, and PCR products with 140–160 bp size were enriched to generate a cDNA library and sequenced using Illumina Xten. Data analysis was performed using the OmicShare tools at www.omicshare.com/tools. The raw miRNA sequencing data generated from this study have been deposited in NCBI GEO (https://www.ncbi.nlm.nih.gov/geo) under the accession number GSE163504.

### Bioinformatics

mRNA expression data of GBM were downloaded from The Cancer Genome Atlas (TCGA, *n* = 162, http://xena.ucsc.edu/getting-started/) and the Chinese Glioma Genome Atlas (CGGA, *n* = 388, http://www.cgga.org.cn) databases. Statistical analyses were performed using Pearson's correlation analysis. Gene set enrichment analysis (GSEA) was performed using GSEA v2.0 software (Broad Institute of MIT, MIT).

### Quantitative Reverse Transcription-Polymerase Chain Reaction (qRT-PCR)

Total RNA was extracted using the TRIzol reagent. For mRNA analysis, cDNA was synthesized from 2 μg total RNA using PrimeScrip RT reagent kit (TaKaRa Biotechnology, Dalian, China). Quantitative PCR was performed on Applied Biosystems 7500 Real-time PCR system using a SYBR Premix Ex Taq Kit (Takara), with β-actin as a reference control. Primers are listed in Supplementary Table 1.

### Western Blotting

Cells were lysed using the radio immunoprecipitation assay (RIPA) buffer (Beyotime, Shanghai, China) containing 10 mM phenylmethanesulfonyl fluoride (PMSF). Protein samples were separated by sodium dodecyl sulfate-12% polyacrylamide gel (SDS-PAGE) electrophoresis, and electro-transferred onto polyvinyl difluoride (PVDF) membranes (Millipore, Billerica, MA). Membranes were blocked with 5% skim milk for 1 h, and incubated with primary antibody at 4°C overnight followed by secondary antibody for 1 h at room temperature. Membranes were developed with enhanced chemiluminescence (ECL, Thermo Fisher) and detected using ChemiDoc Touch Imaging System (BioRad). Antibodies included β-actin (1:2,000, Santa Cruz Biotechnology), HMGB1 (1:1,000, Abcam), CD133 (1:1,000, Abcam), SOX2 (1:1,000, Abcam), OCT4 (1:1,000, Abcam), NANOG (1:1,000, Abcam), TLR2 (1:1,000, CST, Boston, MA), p-GSK-3β (1:1,000, CST), β-catenin (1:1,000, CST), c-MYC (1:1,000, SAB), HRP-conjugated goat anti-rabbit IgG (Genshare, Xian, China) and HRP-conjugated goat anti-mouse IgG (Genshare).

### Subcutaneous Patient-Derived GBM Xenograft Model

All experiments involving mice were approved by the Animal Experiment Administration Committee of the Fourth Military Medical University. 5 × 10^6^ patient-derived GBM cells suspended in 100 μL PBS were inoculated subcutaneously into the right forelimb interior root of BABL/c-A nude mice (female) at 4 weeks of age. About 7–8 days after cell implantation, the mice bearing tumor around 50 mm^3^ were randomly divided into a control group, glycyrrhizin group, TMZ group or TMZ + glycyrrhizin group. Mice in the control group received equivalent drug vehicle (dimethyl sulfoxide, DMSO), mice in glycyrrhizin group received 10 mg/kg glycyrrhizin (SelleckChem, Houston TX, USA) five times per week for 2 weeks (intraperitioneal injection, i.p.), mice in TMZ group received 5 mg/kg TMZ five times per week for 3 weeks (i.p.), and mice in TMZ + glycyrrhizin group received 10 mg/kg glycyrrhizin five times per week for 2 weeks and also 5 mg/kg TMZ five times per week for 3 weeks (i.p.). Tumor volume was measured every 3 days with a caliper and calculated using the formula tumor volume (mm^3^) = (length × width^2^) /2. About 24 days after the first treatment, all mice were euthanized and the tumor were carefully removed, weighed.

### Statistics

All the statistical analyses were performed with Graph Pad Prism 7.0 software. The unpaired and two-tailed Student's *t*-test was used to determine the statistical significance between groups. All data were shown as the mean ± standard error of mean (SEM). *P* < 0.05 was considered statistically significant.

## Results

### TMZ Treatment Promote GSCs Formation in Culture

**Figure 1:**
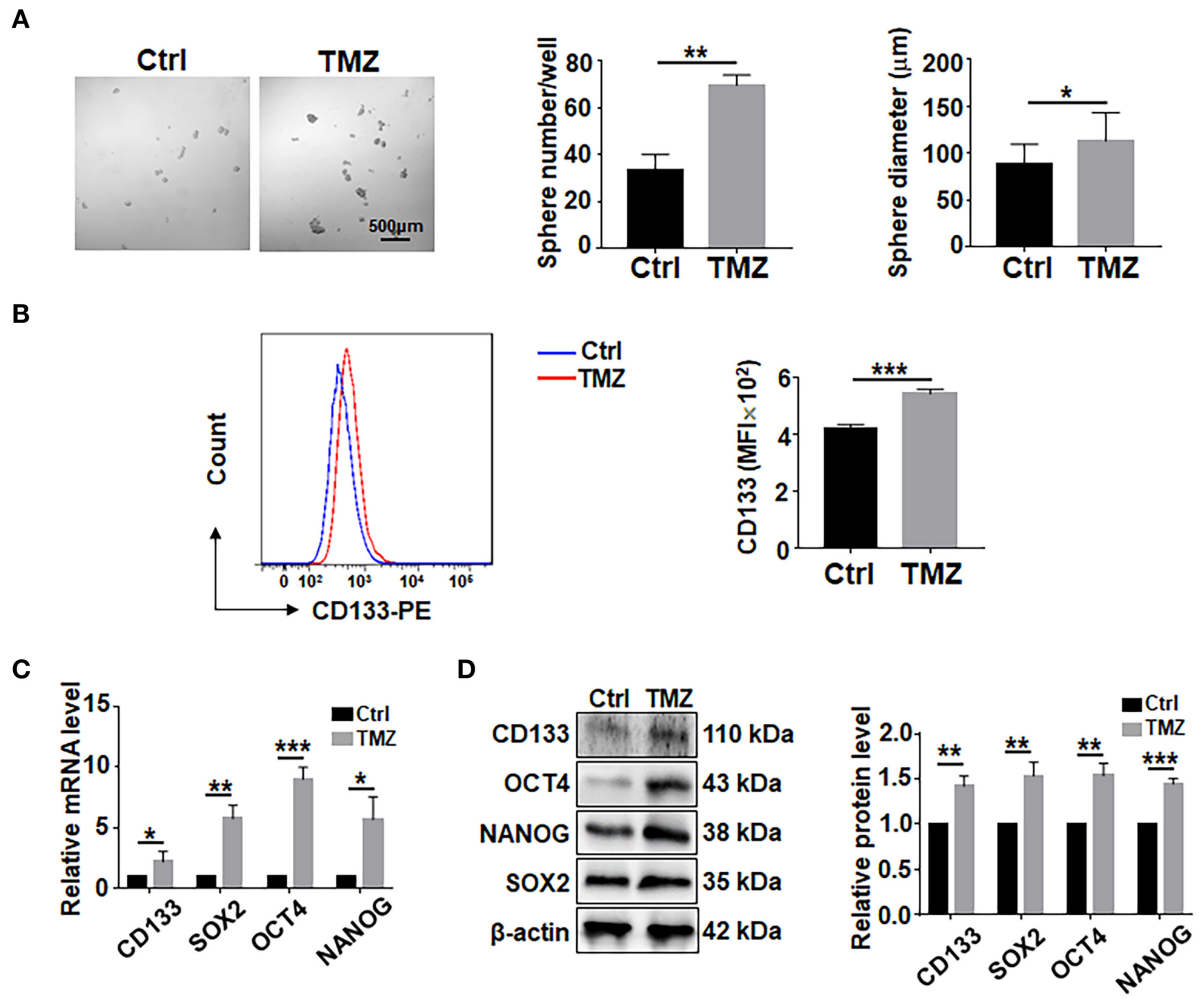
TMZ treatment promote GSCs formation in culture. **(A)** Primary GBM cells were treated with TMZ for 48 h, and then cultured under the neurosphere condition for 7 days and photographed. Number and diameter of spheres on day 7 were quantified. Cell not treated with TMZ were used as controls. **(B)** Primary GBM cells treated with TMZ for 48 h were analyed by flow cytometry for CD133 (*n* = 3), MFI, mean fluorescence intensity. **(C)** Expression of stemness-related factors in primary glioma cells after TMZ stimulation was determined by qRT-PCR (*n* = 3). **(D)** Expression of stemness-related factors in primary glioma cells after TMZ stimulation was determined by western blot (*n* = 3). Data are represented as mean ± SEM, ^*^P < 0.05; ^**^P < 0.01; ^***^P < 0.001.

To assess the effects of TMZ on GBM cells, biopsy-derived GBM cells were treated with TMZ (300 μM) for 48 h, followed by culturing in ultra-low adhesion plates under the neurosphere condition for 7 days. The result showed that TMZ treatment could promote the formation of tumor spheres in terms of sphere number and size (Figure 1A). The results of flow cytometry experiment showed that the surface expression level of CD133 in GBM cells was increased after TMZ treatment (Figure 1B). Analyses of qRT-PCR and western blotting showed that TMZ treatment increased the expression of CD133, SOX2, OCT4, and NANOG (Figures 1C,D), suggesting that TMZ treatment promotes GSCs formation in GBM cells.

### TMZ Upregulates HMGB1 in GBM Cells

**Figure 2:**
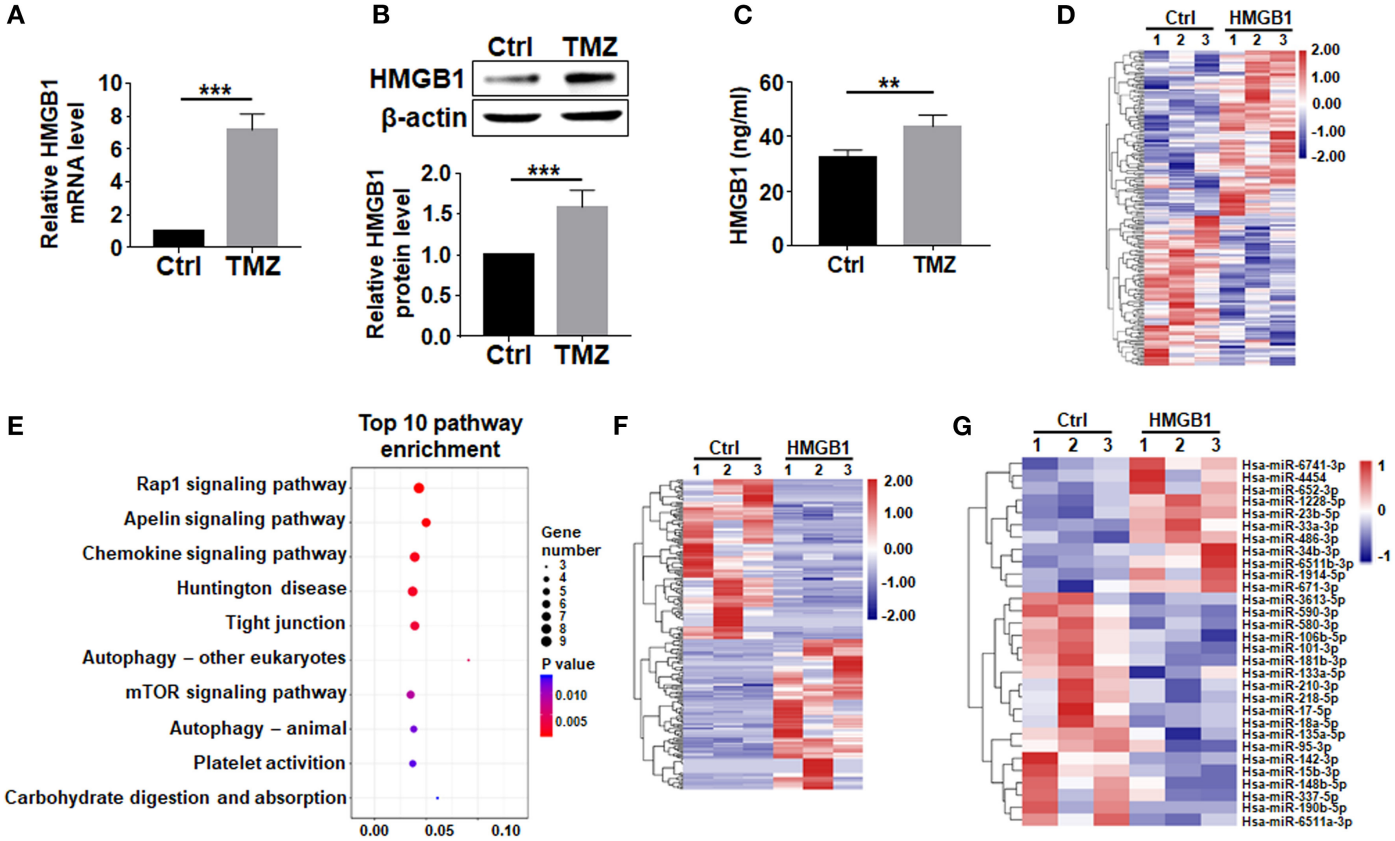
TMZ upregulates HMGB1 in GBM cells. **(A)** Expression of HMGB1 in glioma cells from a single GBM biopsy after TMZ stimulation was determined by qRT-PCR (*n* = 5). **(B)** Expression of HMGB1 in glioma cells from a single GBM biopsy after TMZ stimulation was determined by western blot (*n* = 5). **(C)** ELISA-based analysis of the HMGB1 concentration in the culture supernatants of glioma cells from a single GBM biopsy at 48 h (*n* = 5). **(D)** Heatmap of mRNA-seq analysis from a single GBM biopsy (GBM cells treated with 800 ng/ml rhHMGB1, *n* = 3; control GBM cells, *n* = 3). Two hundred and nineteen genes were identified with a cut-off of >1.2-fold for gene expression change and *p* < 0.05. **(E)** KEGG analysis was performed to identify differential pathway enrichment. **(F)** Heatmap of lncRNA-seq analysis from a single GBM biopsy (GBM cells treated with 800 ng/ml rhHMGB1, *n* = 3; control GBM cells, *n* = 3). Two hundred and twenty-one lncRNAs were identified with a cut-off of >1.2-fold for gene expression change and *p* < 0.05. **(G)** Heatmap of miRNA-seq analysis from a single GBM biopsy (GBM cells treated with 800 ng/ml rhHMGB1, *n* = 3; control GBM cells, *n* = 3). Thirty hsa-miRNAs were identified with a cut-off of >1.2-fold for gene expression change and *p* < 0.05. Data are represented as mean ± SEM, *P < 0.05; **P < 0.01; ***P < 0.001.

HMGB1, a well-known DAMP released by damaged cells, is reported to upregulate pluripotency-related genes in GBM cells (Zang et al., 2020). We then asked whether TMZ treatment could promote the expression of HMGB1. qRT-PCR and western blotting showed that TMZ upregulated HMGB1 in GBM cells at both mRNA and protein levels (Figures 2A,B). While most HMGB1 located in nuclei as visualized by immunofluorescence (Supplementary Figure 1A). The release of HMGB1 protein was significantly increased in the culture supernatants after TMZ treatment (Figure 2C). These results suggested that TMZ treatment induced GBM cell-derived HMGB1 in TME.

To explore the effect of HMGB1 on GBM cells, we compared transcriptomes of GBM cells treated with rhHMGB1 (800 ng/ml, 48 h) with the control (PBS-treated) by RNA-seq. Our result showed that HMGB1 treatment did not lead to dramatically transcriptional changes in GBM cells, as suggested by the principal component analysis (PCA) (Supplementary Figure 1B). However, a total of 115 upregulated and 104 downregulated encoding genes were still detected in GBM cells treated with HMGB1 (Figure 2D). Gene pathway enrichment analysis displayed pathways that are potentially activated by HMGB1 (Figure 2E), some of which have been demonstrated in experiments reported previously (Lin et al., 2016; Meng et al., 2018; Xu et al., 2019). We also identified 107 upregulated and 114 downregulated lncRNAs, as well as a group of differentially expressed miRNAs in GBM cells after HMGB1 treatment (Figures 2F,G). These results suggested that GBM cells are targets of TME-derived HMGB1.

### HMGB1 Promotes the Formation of GSCs

**Figure 3:**
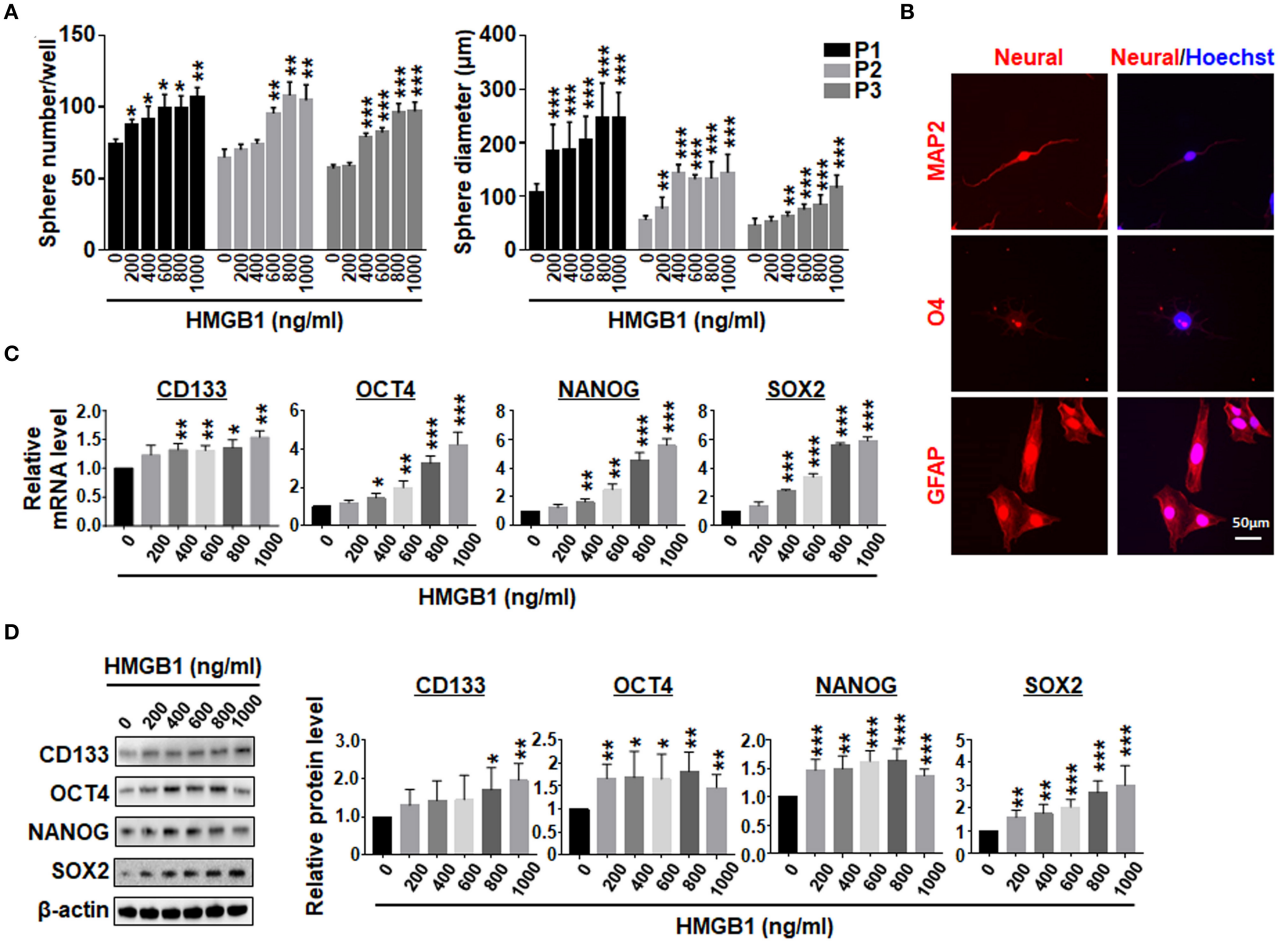
HMGB1 promotes the formation of GSCs. **(A)** Primary GBM cells were cultured under the neurosphere condition with different concentration of HMGB1 for 7 days (P1). The tumor spheres were re-plated every 7 days for another 2 passages (P2, 3). Number and diameter of tumor spheres on day 7 were quantified. **(B)** Tumor spheres derived from HMGB1-induced or control primary glioma cells were subjected to differentiation culture for 7 days. The expression of markers of neurons (MAP-2), oligodendrocytes (O4) and astrocytes (GFAP) were determined by immunofluorescence, and counter-stained with Hoechst. **(C)** Expression of stemness-related factors in primary glioma cells treated with different concentration of HMGB1 was determined by qRT-PCR (*n* = 3). **(D)** Expression of stemness-related factors in primary glioma cells treated with different concentration of HMGB1 was determined by western blot (*n* = 5). Data are represented as mean ± SEM, *P < 0.05; **P < 0.01; ***P < 0.001.

GSEA analysis showed that HMGB1 upregulated pluripotency-related genes in GBM cells (Supplementary Figures 1C,D), consistent with previously reports (Zang et al., 2020). We then cultured GBM cells with different concentrations of HMGB1, and determined the formation of tumor spheres under the neural sphere culture condition. The result showed that the number and size of tumor spheres increased proportionally with increasing HMGB1 concentrations (Figure 3A; Supplementary Figure 1E). Re-plating assay showed that the number and the size of tumor spheres increased consistently in different passages (Figure 3A; Supplementary Figure 1E). To confirm the stemness of tumor spheres derived from GBM cells stimulated by HMGB1, we cultured dispersed tumor spheres adherently in the presence of serum. The result of immunofluorescence showed that these tumor spheres were able to differentiate into MAP2^+^ neurons, O4^+^ oligodendrocytes and GFAP^+^ astrocytes (Figure 3B). In addition, qRT-PCR and western blotting showed that the expression of GSCs marker CD133 and pluripotency factors including SOX2, OCT4 and NANOG were upregulated proportionally in GBM cells treated with increasing HMGB1 concentrations (Figures 3C,D). Analysis of GBM data from TCGA and CGGA databases showed that HMGB1 expression was positively correlated with CD133, SOX2 and OCT4 expression (Supplementary Figure 2A). These results indicated HMGB1 promotes GSCs formation in cultured GBM cells.

### TMZ Promote GSCs Formation by Upregulating HMGB1

**Figure 4:**
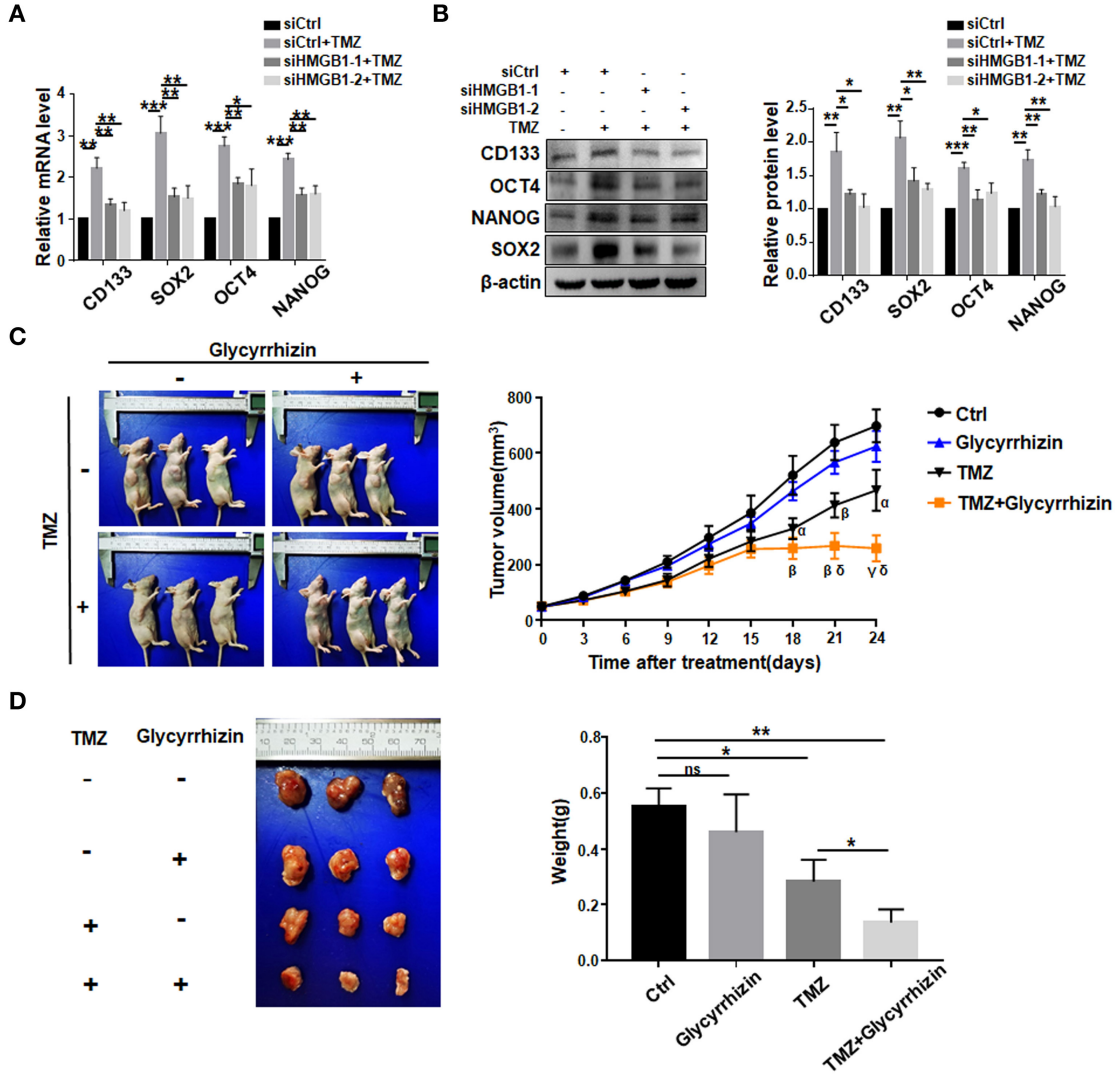
TMZ promote GSCs formation by upregulating HMGB1. **(A,B)** qRT-PCR and western blot analysis of the expression level of stemness-related factors in primary GBM cells co-cultured with supertanant from the following cells: Ctrl siRNA; Ctrl siRNA + TMZ (300 μM); HMGB1 siRNA + TMZ (300 μM). **(C)** Images of subcutaneously xenograft glioma models in nude mice at day 24 after first treatment. Vivo tumor volume was measured every 3 days and the same day collected data were compared among groups. α < 0.05, β < 0.01, γ < 0.001, compared to control group; δ < 0.05, compared to TMZ group. **(D)** Images of glioma tumors taken from the mice of each group and the weight of the tumor is measured at day 24 after first treatment. Data are represented as mean ± SEM, *P < 0.05; **P < 0.01; ***P < 0.001.

We then asked whether TMZ treatment could promote GSCs formation by upregulating HMGB1. We transfected GBM cells with HMGB1 or Ctrl siRNAs (Supplementary Figures 2B,C), and collected culture supernatants. GBM cells were cultured with supernatants derived from siCtrl- or siHMGB1-transfected GBM cells in the presence of TMZ for 48 h. The results of qRT-PCR and western blotting showed that supernatant from siCtrl-transfected GBM cells could promote the expression of CD133, SOX2, OCT4, and NANOG, compared to siHMGB1-transfected GBM cells (Figures 4A,B). The *in vivo* effect of TMZ induced HMGB1 was further explored by xenograft GBM models in nude mice. Glycyrrhizin, a direct HMGB1 inhibitor (Mollica et al., 2007), and TMZ were injected in nude mice with tumors as described above. Compared with the control group, TMZ group had much smaller tumor volume since day 18 after first treatment and this difference became more obvious over time. Average tumor volume of the TMZ + glycyrrhizin group was strikingly smaller than TMZ group since day 21 (Figure 4C). Consistent with alternation in tumor volume, the average tumor weights at the end of vivo experiment in TMZ group were much smaller than that of the control group and greatly larger than that of TMZ + glycyrrhizin group (Figure 4D). The data of vivo experiment indicated that the combination of TMZ and glycyrrhizin exerted a much stronger growth-inhibitory effect on patient-derived GBM xenograft models. In addition, we analyzed the published sequencing data of glioblastoma treated with TMZ and compared them with our sequencing data (Chen et al., 2017; Li et al., 2018; Huang K. et al., 2019; Guo et al., 2020). We found that there were 35 protein-coding genes and 9 miRNAs (miR-23, miR-34, miR-106, miR-142, miR-148, miR-580, miR-590, miR-652 and miR-4454) in the two sets of sequencing data that exhibited similar alterations (Supplementary Figure 3A). Furthermore, 11 out of the top 50 signaling pathways displayed overlapping activity in the two sets of data as the shown KEGG analysis (Supplementary Figure 3B). These results were consistent with that glioblastoma cells released HMGB1 into extracellular space after TMZ treatment and HMGB1 in TME could increase the formation of GSCs.

### TLR2 Mediates HMGB1-Induced GSCs Formation

**Figure 5:**
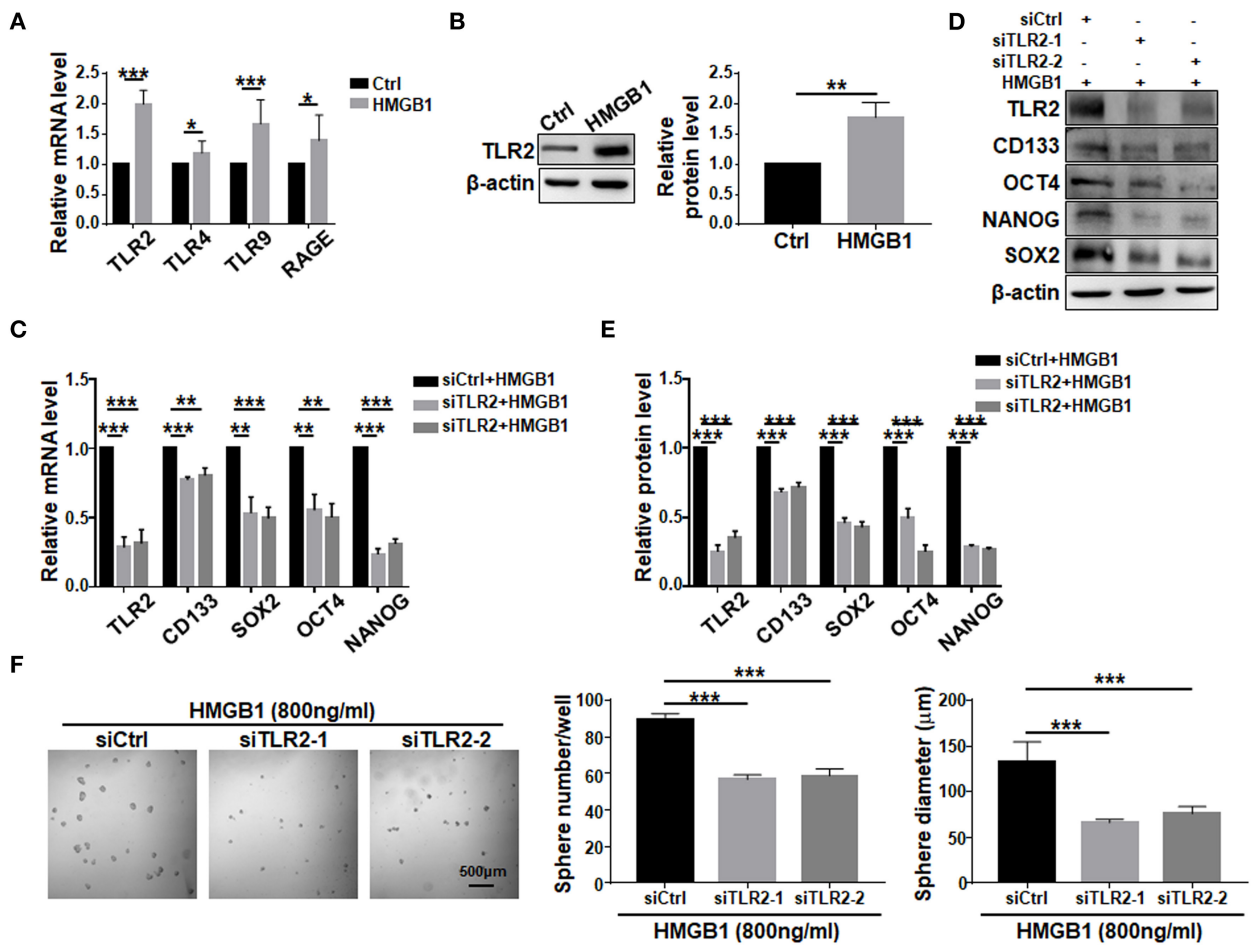
TLR2 is required for HMGB1-induced GSCs formation. **(A)** Expression of TLR2, TLR4, TLR9, RAGE in primary glioma cells after HMGB1 stimulation (800 ng/ml) was determined by qRT-PCR (*n* = 3). **(B)** Expression of TLR2 in primary glioma cells after HMGB1 stimulation (800 ng/ml) was determined by western blot (*n* = 3). **(C)** qRT-PCR analyses of the levels of CD133, OCT4, SOX2, and NANOG in TLR2 knockdown primary GBM cells treated with 800 ng/ml rhHMGB1 (*n* = 3). **(D,E)** Western blot analyses of the levels of CD133, OCT4, SOX2, and NANOG in TLR2 knockdown primary GBM cells treated with 800 ng/ml rhHMGB1 (*n* = 3). **(F)** Primary glioma cells were treated with HMGB1 (800 ng/ml) and transfected with siRNAs to TLR2, and then cultured under the neurosphere condition for 7 d. Number and diameter of tumor spheres on day 7 were quantified. Data are represented as mean ± SEM, *P < 0.05; **P < 0.01; ***P < 0.001.

To investigate signaling pathways mediating HMGB1-induced GSCs formation. TLR2, TLR4, TLR9, and RAGE are the most common receptors of HMGB1 and have been identified in GBM cells (Angelopoulou et al., 2016). Because HMGB1 has been shown to exhibit autocrine activity, we examined the effect of HMGB1 on the expression of different receptors in GBM cells. qRT-PCR analyses showed that TLR2, TLR4, TLR9 and RAGE were accumulated in GBM cells treated with HMGB1 at a concentration of 800 ng/ml, and the expression of TLR2 increased most remarkably (Figure 5A). Consistently, the protein level of TLR2 was upregulated when GBM cells were treated with HMGB1 (Figure 5B). In addition, in gene expression profiling of GBM reported in TCGA and CGGA databases, higher levels of TLR2 correlated with a decrease in median survival of GBM patients and TLR2 was positively correlated with the expression of CD133, SOX2 and OCT4 (Supplementary Figures 4A,B), consistent with previous studies (Chen et al., 2019). We therefore focused our study on TLR2 and siRNAs targeting TLR2 were synthesized. Then we stimulated GBM cells, which were transfected with TLR2 siRNAs, by HMGB1. The results indicated that the mRNA and protein levels of CD133, SOX2, OCT4, and NANOG were downregulated when TLR2 was knocked down (Figures 5C–E). Consistently, the number and the size of tumor spheres decreased significantly by TLR2 siRNAs compared with the negative control (Figure 5F). Our data suggested that HMGB1 might promote GSCs formation via TLR2.

### HMGB1 Promotes GSCs Formation via Wnt/β-Catenin Downstream to TLR2

**Figure 6:**
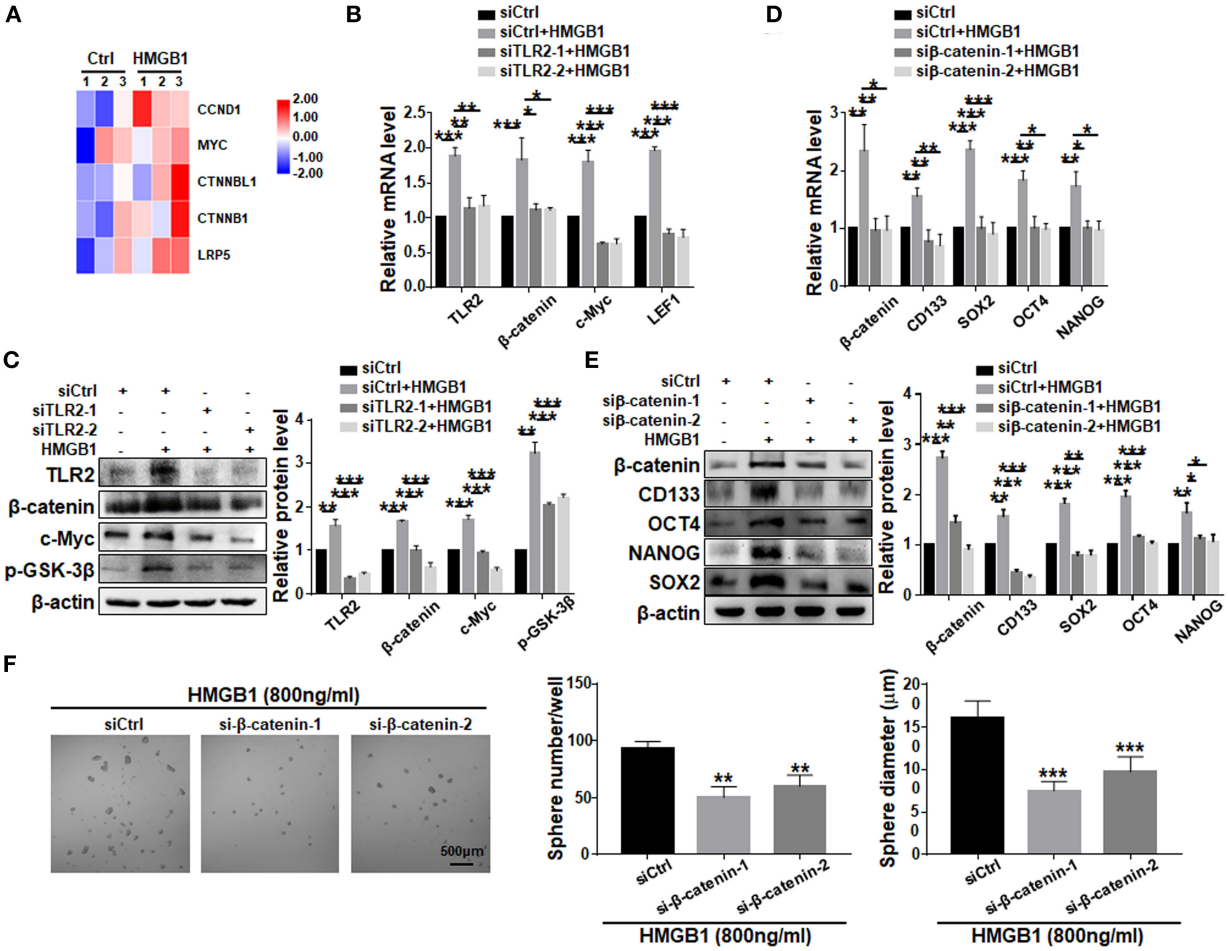
HMGB1 promotes GSCs formation via the Wnt/β-catenin pathway downstream to TLR2. **(A)** Patient derived GBM cells treated with HMGB1 (800 ng/ml) and control GBM cells were subjected to RNA-seq. The expression of Wnt-associated genes is shown by a heatmap. **(B)** qRT-PCR analyses of the levels of TLR2, β-catenin, c-Myc and LEF1 in TLR2 knockdown primary GBM cells treated with 800 ng/ml rhHMGB1 (*n* = 3). **(C)** Western blot analyses of the levels of TLR2, β-catenin, c-Myc and p-GSK-3β in TLR2 knockdown primary GBM cells treated with 800 ng/ml rhHMGB1 (*n* = 3). **(D,E)** qRT-PCR and western blot analyses of the levels of β-catenin, CD133, OCT4, SOX2, and NANOG in β-catenin knockdown primary GBM cells treated with 800 ng/ml rhHMGB1 (*n* = 3). **(F)** Primary glioma cells were treated with HMGB1 (800 ng/ml) and transfected with siRNAs to β-catenin, and then cultured under the neurosphere condition for 7 d. Number and diameter of tumor spheres on day 7 were quantified. Data are represented as mean ± SEM, *P < 0.05; **P < 0.01; ***P < 0.001.

To explore the potential pathway downstream to HMGB1-TLR2, we compared transcriptomes of GBM cells treated with HMGB1 and PBS. Bioinformatic analyses showed that several important molecules in the Wnt pathway were up-regulated, suggesting that Wnt/β-catenin pathway, which plays an important role in the regulation of stemness and tumorigenicity of GSCs (Gong and Huang, 2012), might be activated (Figure 6A). We then knocked down TLR2 in GBM cells in the presence of HMGB1, and determined the expression of Wnt/β-catenin signaling-related molecules by qRT-PCR and western blotting. The results confirmed that the expression of β-catenin, c-Myc, p-GSK-3β and LEF1 were upregulated in GBM cells in presence of HMGB1, and silencing TLR2 by siRNAs could attenuate this effect (Figures 6B,C), suggesting that HMGB1 activated Wnt signaling via TLR2. To further explore the role of Wnt/β-catenin signaling pathway in formation of GSCs in HMGB1-treated GBM cells, we synthesized siRNAs targeting β-catenin and transfected GBM cells stimulated by HMGB1. Analyses of qRT-PCR and western blotting showed that the expression of CD133, SOX2, OCT4 and NANOG were down-regulated by β-catenin siRNAs in the presence of HMGB1 (Figures 6D,E). Consistently, the number and the size of tumor spheres were attenuated significantly by β-catenin siRNAs compared with the negative control (Figure 6F). These results suggested that HMGB1-TLR2 promote GSCs formation by activating the Wnt/β-catenin signaling pathway.

### LncRNA NEAT1 Is Required for the Formation of HMGB1 Induced GSCs

**Figure 7:**
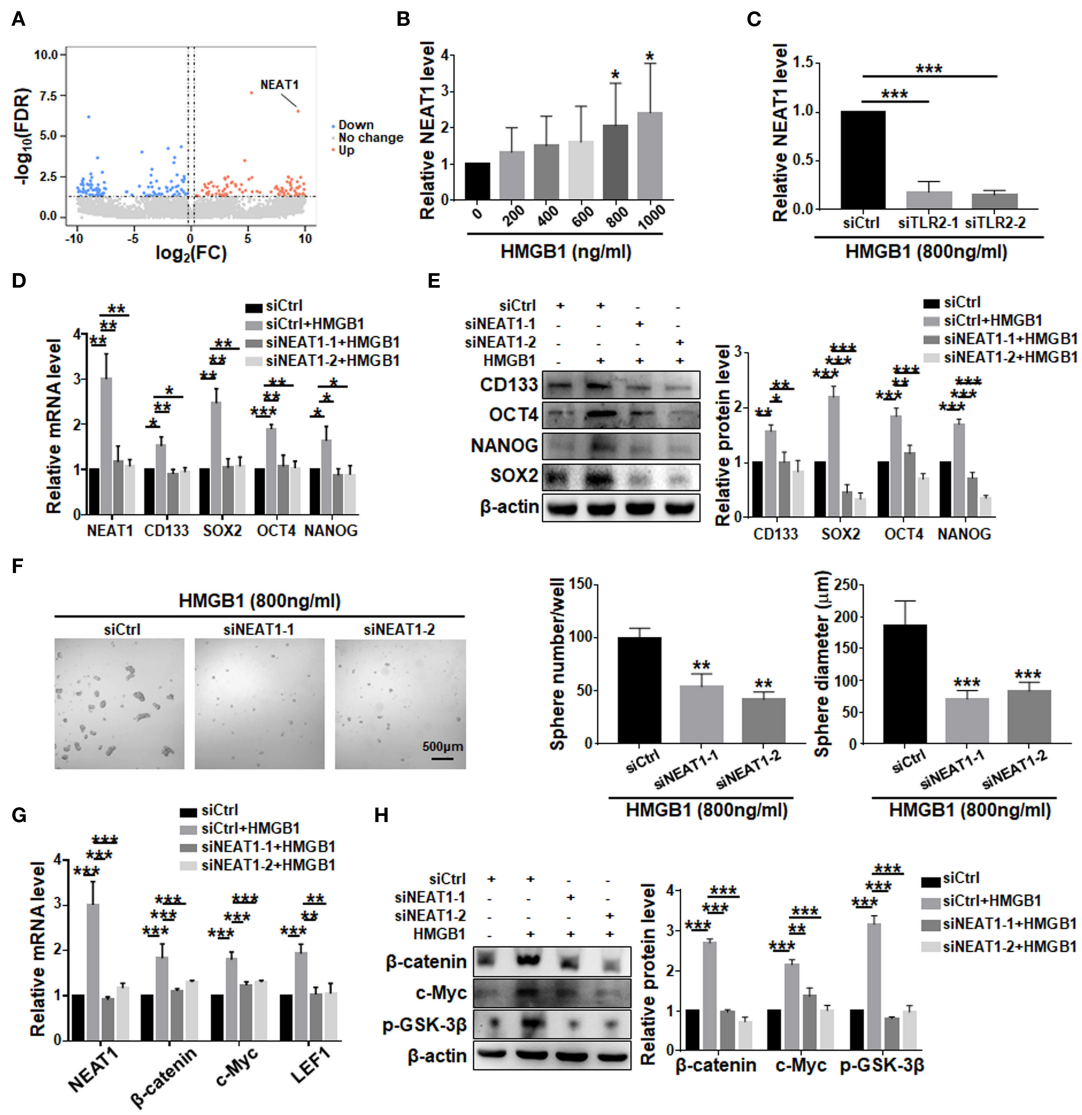
LncRNA NEAT1 is required for the formation of HMGB1 induced GSCs. **(A)** Patient derived GBM cells treated with HMGB1 (800 ng/ml) and control GBM cells were subjected to lncRNA-seq. The expression level is shown by a heatmap. **(B)** Expression of NEAT1 in primary glioma cells treated with different concentration of HMGB1 was determined by qRT-PCR (*n* = 6). **(C)** qRT-PCR analyses of the levels of NEAT1 in TLR2 knockdown primary GBM cells treated with 800 ng/ml rhHMGB1 (*n* = 3). **(D,E)** qRT-PCR and western blot analyses of the levels of NEAT1, CD133, SOX2, OCT4 and NANOG in NEAT1 knockdown primary GBM cells treated with 800 ng/ml rhHMGB1. **(F)** Primary glioma cells were treated with HMGB1 (800 ng/ml) and transfected with siRNAs to NEAT1, and then cultured under the neurosphere condition for 7 d. Number and diameter of tumor spheres on day 7 were quantified. **(G)** qRT-PCR analyses of the levels of NEAT1, β-catenin, c-Myc and LEF1 in NEAT1 knockdown primary GBM cells treated with 800 ng/ml rhHMGB1 (*n* = 3). **(H)** Western blot analyses of the levels of β-catenin, c-Myc and p-GSK-3β in NEAT1 knockdown primary GBM cells treated with 800 ng/ml rhHMGB1 (*n* = 3). Data are represented as mean ± SEM, *P < 0.05; **P < 0.01; ***P < 0.001.

Comparison of transcriptomes of GBM cells treated with HMGB1 and PBS showed that the expression of NEAT1, a lncRNA reportedly playing important roles in GSCs (Gong et al., 2016; Yang et al., 2017; Lulli et al., 2020), was significantly upregulated (Figure 7A). qRT-PCR confirmed the proportional upregulation of NEAT1 in the presence of increasing concentrations of HMGB1 (Figure 7B). Silencing TLR2 by siRNA reduced the expression of NEAT1 in the presence of HMGB1 (Figure 7C). To confirm a potential role of NEAT1 in HMGB1-induced GSCs, we synthesized siRNA targeting NEAT1. The results showed that silencing NEAT1 by siRNAs reduced the mRNA and protein levels of CD133, SOX2, OCT4 and NANOG (Figures 7D,E). The number and size of tumor spheres decreased also upon NEAT1 knockdown (Figure 7F). We conclude that NEAT1 is required for HMGB1-induced GSCs formation. In addition, silencing NEAT1 by siRNA abrogated upregulation of β-catenin, c-Myc, p-GSK-3β and LEF1, as shown by qRT-PCR and western blotting, respectively (Figures 7G,H). Therefore, NEAT1 is downstream to TLR2 and activates Wnt/β-catenin in GBM cells to promote GSCs formation.

## Discussion

CSCs or tumor-initiating cells are considered as drivers of tumor growth and relapse, and are often identified in heterogeneous, aggressive and therapy-resistant tumors (Walcher et al., 2020). Although the mechanisms of CSCs for tumor growth, recurrence, and drug resistance have been well documented, the origin of CSCs is not entirely clear. One opinion suggests that CSCs can be generated under the pressure of chemotherapy and changes in TME, in other words, CSCs could originate from non-CSC tumor cells (De Angelis et al., 2019). GBM is the most common malignant tumor in the brain and there have been many reports demonstrating the existence of GSCs in GBM (Varghese et al., 2008). In the current study, we show that GBM-derived HMGB1 promote the formation of GSCs from patient-derived GBM cells treated with TMZ. Chemotherapy could cause ICD of tumor cells that release DAMPs into TME. DAMPs induced by ICD include heat shock protein 70 (HSP70), calreticulin, ATP and HMGB1 (Garg et al., 2015). TMZ could elevate the secretion of HSP70, calreticulin, ATP and HMGB1 in TME (Liikanen et al., 2013; Pasi et al., 2014). It has been reported that HSP70 and calreticulin are beneficial for glioma patients, while ATP could serve as a critical signaling molecule supporting glioblastoma growth (Jantaratnotai et al., 2009; Muth et al., 2016). Our results showed that TMZ could up-regulate the expression of HMGB1 in primary GBM cells and promote the release of HMGB1 into TME after TMZ treatment. Previous studies have suggested that extracellular HMGB1 could enhance and maintain the stemness of CSCs in breast cancer, colorectal cancer and pancreatic cancer (Zhao et al., 2017; Qian et al., 2019; Zhang et al., 2019). Our study demonstrated for the pivotal role of HMGB1 in promoting GSCs formation in patient-derived primary glioma cells upon TMZ treatment. In addition, we analyzed the published sequencing data of glioblastoma treated with TMZ and compared them with our sequencing data (Chen et al., 2017; Li et al., 2018; Huang K. et al., 2019; Guo et al., 2020). We found that 35 protein-coding genes and nine miRNAs exhibited similar alterations in the two sets of sequencing data. Furthermore, 11 out of the top 50 signaling pathways displayed overlapping activity in the two sets of data as the shown KEGG analysis. These results further confirmed that glioblastoma cells released HMGB1 into extracellular space after TMZ treatment and HMGB1 in TME could increase the formation of GSCs. Serum HMGB1 level is quite low in glioma patients untreated or treated with TMZ (Liikanen et al., 2013; Kluckova et al., 2020), in contrast with HMGB1 in culture in our experiments. However, serum HMGB1 level could not represent HMGB1 in TME. It has been reported that HMGB1 expression was significantly higher in TME than in adjacent non-tumor tissues (Cheng et al., 2018). Referring to other published literatures (varying between 150 and 2,000 ng/ml. For instance, Zhao et al., 2017; Chen et al., 2019), we examined the effect of different concentrations of HMGB1 on GSCs, and used 800 ng/ml in most of our experiments, because with this concentration, HMGB1 induced significant stemness in glioma cells. To examine the role of HMGB1 *in vivo*, we established subcutaneous GBM xenograft models with patient-derived GBM cells, and observed the effect of glycyrrhizin, a HMGB1 inhibitor, on tumor growth. The results showed that glycyrrhizin reinforced the growth-inhibitory effect of TMZ on xenograft GBM, suggesting that HMGB1 plays an important role in promoting GBM growth in the presence of TMZ. Briefly, our observations possess clinical significance, because recurrence of glioma after TMZ treatment has always been an urgent challenge in clinical treatment of GBM patients.

Currently, the clinical use of TMZ is 150–200 mg/mm^2^. In *in vitro* experiment, considering drug absorption and metabolism, we could not directly use the *in vivo* dosage. The TMZ dosage we used in our experiments was the one that induces glioblastoma cell death in dose- and time-dependent manners *in vitro* (Chen et al., 2017; Huang W. et al., 2019). Glycyrrhizin, a direct HMGB1 inhibitor, reportedly could exert inhibitory effects on the proliferation of human glioblastoma U251 cell line (Li et al., 2014). Glycyrrhizin has been tested in the treatment of various diseases, such as psoriasis and vitiligo (Yu et al., 2017; Li et al., 2019), but not in the glioma. Our *in vivo* result showed that glycyrrhizin reinforced the growth-inhibitory effect of TMZ on xenograft GBM. Our study provided a significant research basis for performing further investigation on glycyrrhizin in glioma therapy and combining TMZ and glycyrrhizin might be considered as a future therapeutic strategy.

After being released into TME, HMGB1 needs to interact with its high-affinity receptors to elicit its biological functions. TLR2, TLR4, TLR9, and RAGE have been identified as the most common receptors for HMGB1 on cell surface in different cancer models and patients (Angelopoulou et al., 2016; Qian et al., 2019; Zhang et al., 2019). The effects of activating TLR2 on glioma cells are complicated and sometimes contradictory. Wang et al. reported that activation of TLR2 promotes tumor invasion by upregulating MMPs in glioma stem cells (Wang F. et al., 2015). Another study by Curtin et al. indicated that TLR2 activation could promote glioma regression (Curtin et al., 2009). According to our findings, TLR2 functions as the major receptor responsible for HMGB1-mediated GSCs formation in patient-derived GBM cells. TLR2 has been demonstrated to affect cancer cell behaviors by activating several downstream signaling pathways, including NF-kB, PI3K/Akt and Wnt/β-catenin pathways (Liu et al., 2018; Chen et al., 2019). Our results have shown that TLR2 participates in GSCs formation most likely via Wnt/β-catenin signaling, which is a classic pathway regulating the pluripotency of stem cells and determines the fate of cell differentiation during development.

The nuclear paraspeckle assembly transcript 1 (NEAT1) is a long non-coding RNA, and is often highly expressed in human tumors with different origins. Clinical studies have shown that patients with high NEAT1 expression have a poor prognosis (Pan et al., 2015; Chen et al., 2016; Han et al., 2018). NEAT1 drives the occurrence and development of tumors by regulating genes associated with tumor cell growth, migration, invasion, stem-like phenotypes, and chemotherapeutic and radiological resistance. These characteristics indicate that NEAT1 has the potential to be a new diagnostic biomarker and therapeutic target (Dong et al., 2018). Additionally, NEAT1 is reported to be overexpressed in GSCs, and silencing the expression of NEAT1 in GSCs could weaken their capacity of proliferation, invasion and migration (Yang et al., 2017). In lung cancer cell lines, down-regulation of NEAT1 could decrease the expression of stemness-related factors, including CD133, CD44, SOX2, OCT4 and NANOG (Jiang et al., 2018). In our study, we have shown that NEAT1 is upregulated in GBM cells after HMGB1 stimulation, and knocking down NEAT1 could abrogate HMGB1-induced upregulation of stemness-related factors and GSCs formation. Mechanistically, some TME-derived signals, such as hypoxia, and activation of STAT3 and NF-κB induced by EGFR signaling could promote NEAT1 expression (Choudhry et al., 2015; Chen et al., 2018). In addition, Chen et al. reported that NEAT1 overexpression could induce the activity of the Wnt/β-catenin signaling to mediate tumorigenesis and progression in GBM (Chen et al., 2018). Consistently, our data have shown that upregulation of NEAT1 in GSCs in the presence of HMGB1 could be attributed to TLR2 activation, and NEAT1 could activate Wnt/β-catenin signaling to promote GSCs formation (Supplementary Figure 4C).

In conclusion, we have found that patient-derived GBM cells release HMGB1 into extracellular space after TMZ treatment, and HMGB1 in TME could increase the formation of GSCs, which further induce TMZ resistance. Mechanistically, HMGB1 upregulates lncRNA NEAT1, which is downstream to TLR2 and plays pivotal roles in GSCs formation likely by activating Wnt/β-catenin signaling. Our results provide a new potential strategy to overcome TMZ resistance in GBM patients. Combining TMZ and an HMGB1 inhibitor may be considered as a future therapeutic strategy.

## Data Availability Statement

The datasets presented in this study can be found in online repositories. The names of the repository/repositories and accession number(s) can be found in the article/Supplementary Material.

## Ethics Statement

The studies involving human participants were reviewed and approved by Ethics Committee of Xijing Hospital. The patients/participants provided their written informed consent to participate in this study. The animal study was reviewed and approved by Ethics Committee of Xijing Hospital.

## Author Contributions

X-YG performed the experiments and wrote the manuscript. JZ, M-HZ, Y-FZ, and K-YY collected the data. HH, X-FJ, and LL designed the project. YC, X-XL, and X-LC analyzed the data. LL, X-XL, and HH revised the manuscript. All authors contributed to the article and approved the submitted version.

## Conflict of Interest

The authors declare that the research was conducted in the absence of any commercial or financial relationships that could be construed as a potential conflict of interest.
