# Supplementary material for: Temozolomide Treatment Induces HMGB1 to Promote the Formation of Glioma Stem Cells via the TLR2/NEAT1/Wnt Pathway in Glioblastoma
Source: Front Cell Dev Biol. 2021 Feb 1;9:620883. doi: 10.3389/fcell.2021.620883 (PMC7891666; doi:10.3389/fcell.2021.620883)
Supplement: Supplementary file 1 [file Table_1.DOCX]

Supplementary Material

## Supplementary Table S1. Primer sequences used for qRT-PCR analysis.

| Gene | Forward | Reverse |
| --- | --- | --- |
| β-actin | 5’- AGAAAATCTGGCACCACACC-3’ | 5’-AGAGGCGTACAGGGATAGCA-3’ |
| CD133 | 5’-AGTGGCATCGTGCAAACCTG-3’ | 5’-CTCCGAATCCATTCGACGATAGTA-3’ |
| SOX2 | 5'-CACACTGCCCCTCTCAC-3' | 5'-TCCATGCTGTTTCTTACTCTCC-3' |
| OCT4 | 5’-TCTCCCATGCATTCAAACTGAG-3’ | 5’-CCTTTGTGTTCCCAATTCCTTC-3’ |
| NANOG | 5’-GAAATACCTCAGCCTCCAGC-3’ | 5’-GCGTCACACCATTGCTATTC-3’ |
| HMGB1 | 5’-TATGGCAAAAGCGGACAAGG-3’ | 5’-CTTCGCAACATCACCAATGGA-3’ |
| TLR2 | 5’-CCTCTCGGTGTCGGAATGTC-3’ | 5’-TCCCGCTCACTGTAAGAAACA-3’ |
| TLR4 | 5’-TGCGTGAGACCAGAAAGC-3’ | 5’-TTAAAGCTCAGGTCCAGGTTC-3’ |
| TLR9 | 5’-CTGCCACATGACCATCGAG-3’ | 5’-GGACAGGGATATGAGGGATTTGG-3’ |
| RAGE | 5’-GTGTCCTTCCCAACGGCTC-3’ | 5’-ATTGCCTGGCACCGGAAAA-3’ |
| β-catenin | 5’-AAAGCGGCTGTTAGTCACTGG-3’ | 5’-CGAGTCATTGCATACTGTCCAT-3’ |
| c-myc | 5’-AATGAAAAGGCCCCCAAGGTAG-3’ | 5’-GTCGTTTCCGCAACAAGTCCT-3’ |
| LEF1 | 5’-AGAACACCCCGATGACGGA-3’ | 5’-GGCATCATTATGTACCCGGAAT-3’ |
| NEAT1 | 5’-CCAGTTTTCCGAGAACCAAA-3’ | 5’-ATGCTGATCTGCTGCGTATG-3’ |

## Supplementary Figures

**Supplementary Figure 1.** HMGB1 promotes GSC formation. **A.** Immunofluorescence was performed to evaluate the expression of HMGB1 in GBM cells treated with TMZ stimulation (300 μM) for 48 h. Scale bar, 50 μm. **B.** PCA of RNA-seq data from HMGB1-treated and untreated (Ctrl) GBM cells. **C.** GSEA of RNA-seq data. Gene sets from GO related to somatic stem cell population maintenance is shown. **D.** Genes upregulated or downregulate in GBM cells treated with HMGB1 are shown with a Heatmap. **E.** Primary GBM cells were cultured under the neurosphere condition with different concentration of HMGB1 for 7 days and photographed (P1). The tumor spheres were re-plated every 7 days for another 2 passages (P2, P3).

**Supplementary Figure 2.** Analyses of molecules involved in HMGB1-induced GSC formation. **A.** Correlation between HMGB1 expression and CD133, SOX2 and OCT4 expression in human GBM using data from TCGA (n=162) and CGGA (n=388). **B, C.** GBM cells were transfected with siHMGB1s and siCtrl. HMGB1 expression was determined by western blotting and ELISA.

**Supplementary Figure 3.** Comparison of the sequencing data of glioblastoma treated with TMZ and the sequencing data of glioblastoma treated with HMGB1. **A.** 35 genes in the two sets of RNA-seq data had the same change trend. **B.** 11 of the top 50 signaling pathways in the KEGG analysis of the two sets of data displayed overlapping activity. Left part, RNA-seq data of glioblastoma treated with HMGB1. Right part, RNA-seq data of glioblastoma treated with TMZ.

**Supplementary Figure 4.** Analyses of molecules involved in HMGB1-induced GSC formation. **A.** Kaplan-Meier plots of TLR2 expression and GBM patient survival using data in TCGA (n=162) and CGGA (n=388). **B.** Correlation between TLR2 expression and CD133, SOX2 and OCT4 expression in human GBM using data from TCGA (n=162) and CGGA (n=388). **C.** Schematic illustration of TMZ-induced HMGB1 promoting GSCs formation.
