# Supplementary figures and images for: Temozolomide Treatment Induces HMGB1 to Promote the Formation of Glioma Stem Cells via the TLR2/NEAT1/Wnt Pathway in Glioblastoma
Source: Front Cell Dev Biol. 2021 Feb 1;9:620883. doi: 10.3389/fcell.2021.620883 (PMC7891666; doi:10.3389/fcell.2021.620883)

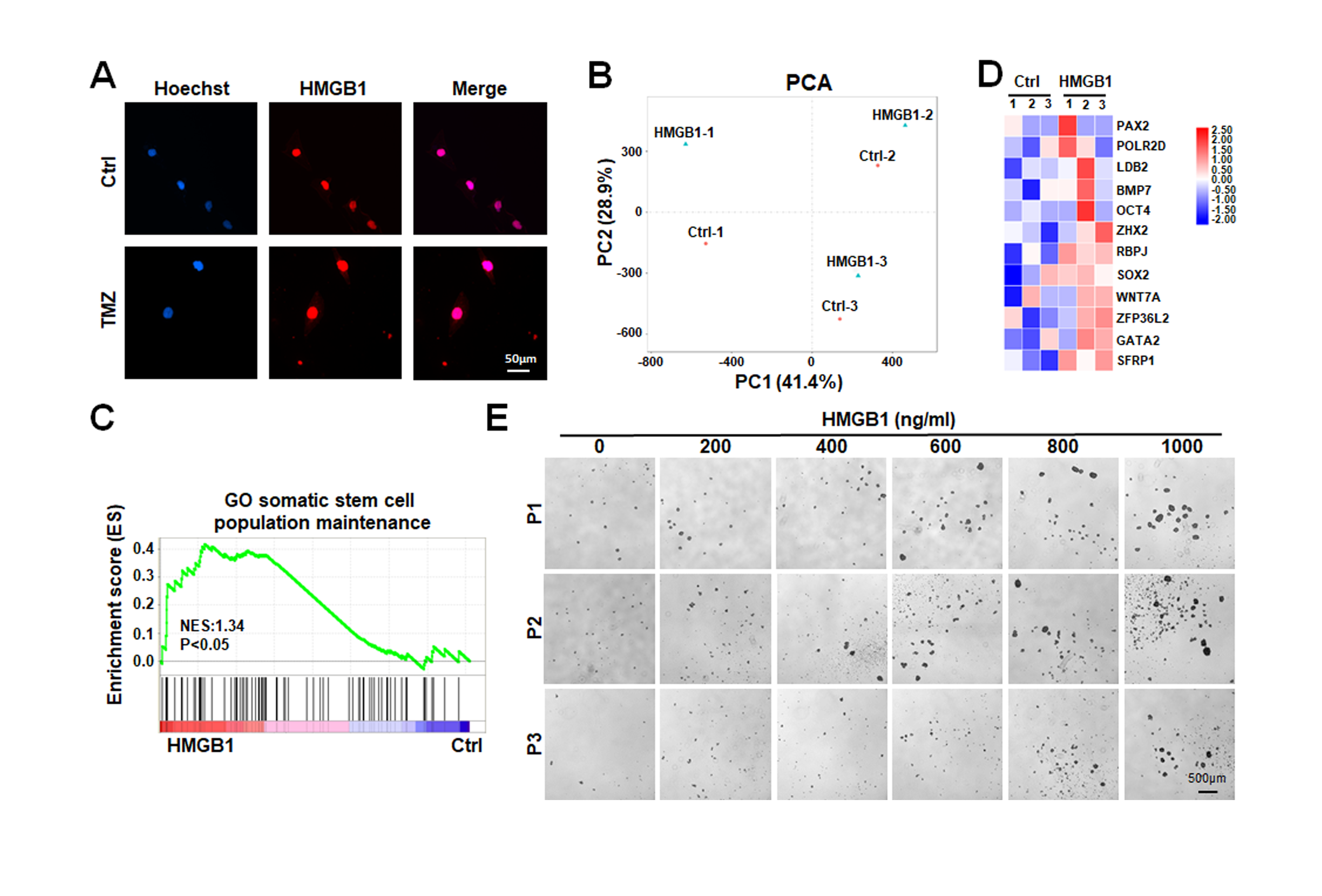

Supplement: Supplementary file 2 [file Image_1.TIF]

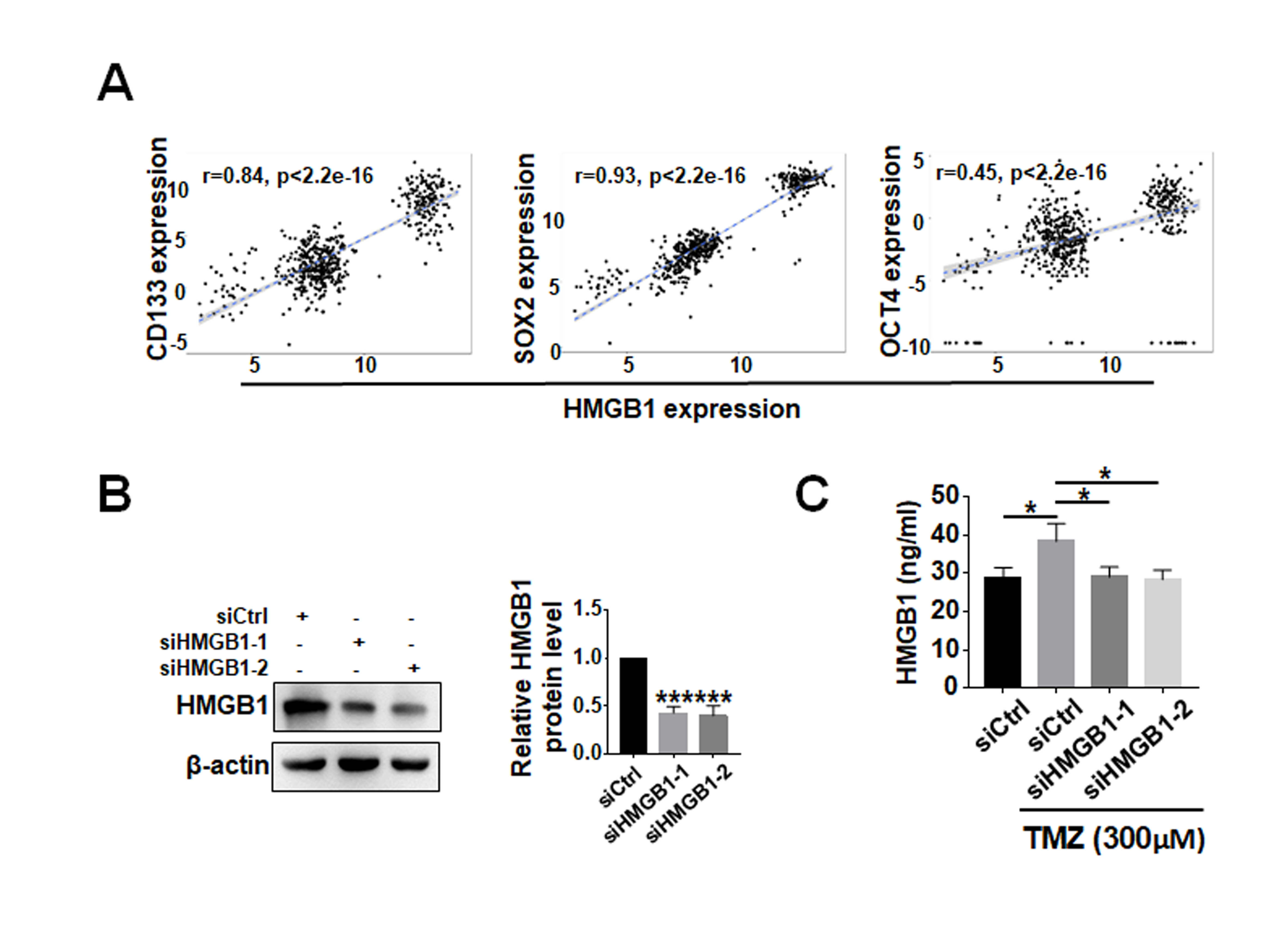

Supplement: Supplementary file 3 [file Image_2.TIF]

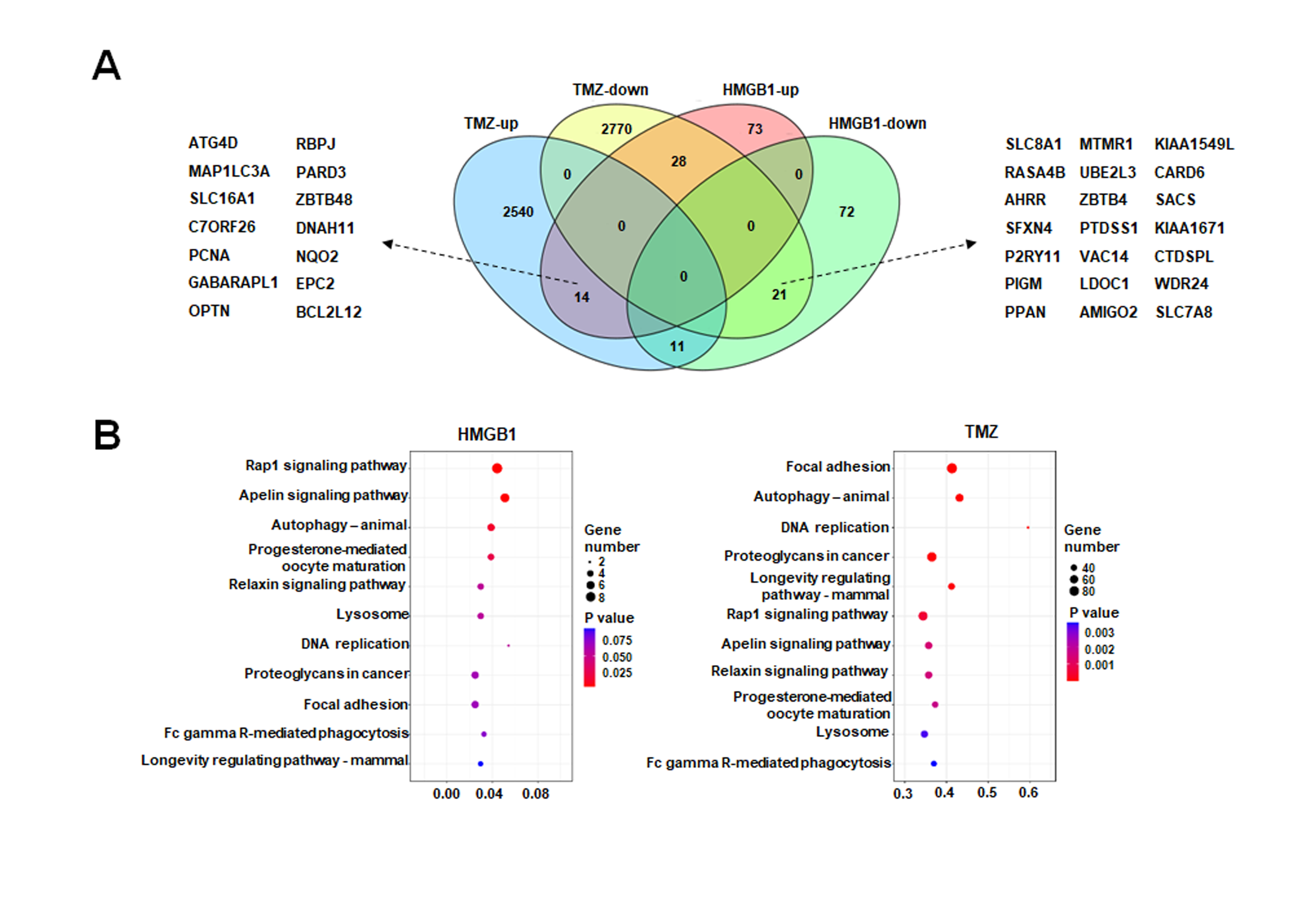

Supplement: Supplementary file 4 [file Image_3.TIF]

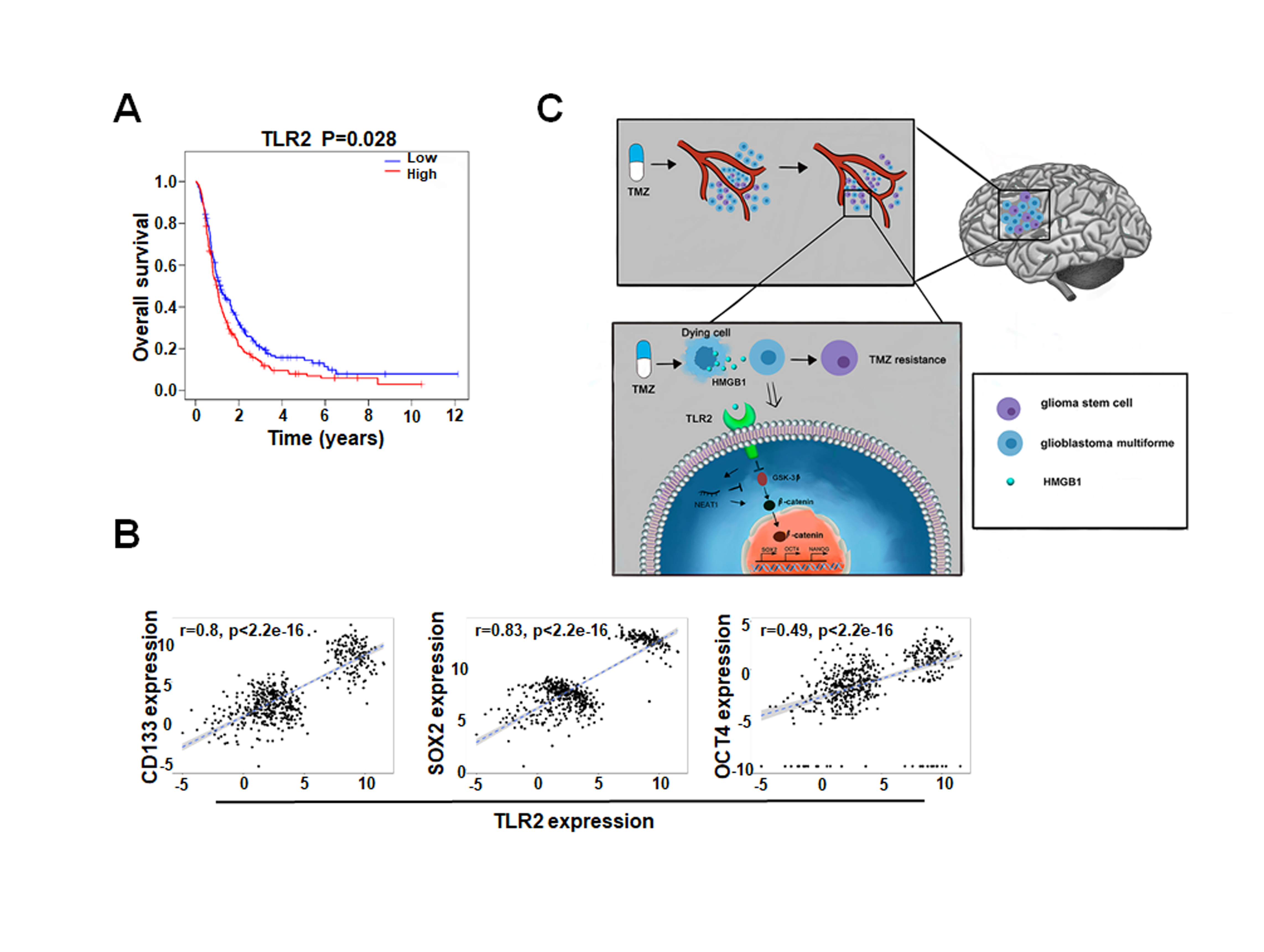

Supplement: Supplementary file 5 [file Image_4.TIF]
